# Expression of PLAGL2 in bladder urothelial carcinoma and its relationship to lymph node metastasis and survival

**DOI:** 10.1038/s41598-018-24526-5

**Published:** 2018-04-16

**Authors:** Genyi Qu, Yong Xu, Shaw P. Wan, Guang Yang

**Affiliations:** 1 Department of Urology, ZhuZhou Central Hospital, South Changjiang Road, Tianyuan district, ZhuZhou, 412007 China; 2 Department of Urology, The First People’s Hospital of Xiaoshan, 199# Xinnan Road, Xiaoshan district, Hangzhou, 311200 China

## Abstract

The purpose of this study was to investigate PLAGL2 expression associated with pathological features and prognosis and predicted lymph node metastases in the bladder urothelial carcinoma (BUC) tissue. The pathologic specimens and clinical data of 203 patients with bladder urothelial carcinoma after radical resection were collected. The expression of PLAGL2 was detected by immunohistochemically staining. The influence on lymph node metastasis and the prognoses of BUC patients were analyzed. The expression of PLAGL2 in BUC and positive lymph nodes was significantly higher than the normal bladder tissues (89.06% and 76.56% vs 21.88%, *P* < 0.001). Logistic regression analysis showed that PLAGL2 expression was an independent risk factor for BUC lymph node metastasis (*P* < 0.05). COX proportional hazards regression model showed that the time to recurrence and overall survival of patients with overexpression of PLAGL2 were significantly lower than those with low expression (*P* < 0.05). PLAGL2 is highly expressed in the BUC tissue and metastatic lymph node relative to the normal bladder tissue. This expression correlates to tumor size and number, and tumor grade and stage. Overexpression of PLAGL2 can be an independent predictor for lymph node metastasis and patient survival.

## Introduction

Each year nearly 430,000 patients are diagnosed with bladder cancer and more than 165,000 patients die from the disease in world-wide^1^. Bladder cancer has become the number one urinary tract cancer in China. The incidence is around 7.68/100,000^2^. Urothelial carcinoma accounts for more than 90% of the bladder cancer. The primary pathway for metastasis is via lymph node; often the first manifestation of bladder cancer is in the pelvic lymph nodes. Lymph node metastasis has a great impact on the treatment strategy and survival of the bladder cancer patients^3^. At present, diagnosis of pelvic lymph node metastasis is mainly based on pelvic MRI and/or CT images. The polymorphic adenoma gene-like protein 2 (PLAGL2) is located on the chromosome 20ql1.21. It contains the initiation codon ATG and the termination codon TAG but devoid of AATAAA polyadenylation signal. Its open reading frame encodes a protein comprising 496 amino acids. This protein contains six zinc finger structures, in an innate sequences in a carboxyl terminal that is rich in rich proline and serine^4^. Many studies have shown that PLAGL2 plays an important role in the development and progression of tumors^5–7^. Yang *et al*.^6^ found that overexpression of PLAGL2 was associated with lung adenocarcinoma and that earlier stages of the disease had a lower expression of PLAGL2. Liu *et al*.^8^ found that the expression of PLAGL2 in the gastric and colorectal cancer tissues was significantly higher than the expression of PLAGL2 in adjacent normal tissues. Moreover, the PLAGL2 expression was positively correlated to the depth of tumor penetration in colorectal cancer; Guo *et al*.^9^ found that PLAGL2 expression in prostate cancer was significantly higher than PLAGL2 expression in benign prostatic hyperplasia tissue. In addition, they found that the extent of PLAGL2 expression in prostate cancer is closely related to the prognoses of patients. PLAGL2 expression can be used as an independent predictor for biochemical recurrence–free survival and OS. All these studies suggested that PLAGL2 plays a role in tumor penetration, metastasis and eventual patient survival. Although the expression and carcinogenic mechanisms of PLAGL2 have been extensively studied, the role of PLAGL2 in the development and progression of bladder cancer is unknown. Therefore, we decided to study the expression of PLAGL2 in bladder cancer. We analyzed the relationship between the expression of PLAGL2 and the clinicopathological features and the prognoses of bladder cancer. This study has been approved by the Institutional Ethics Committee of the Zhuzhou Central Hospital.

## Results

### General information of the patients

A total of 203 patients were entered into this study. Cohorts’ age, gender, tumor size, multiplicity of tumors, tumor grade, cT, and LNS are listed in Table 1.

**Table 1:** Clinical and pathological parameters of 203 patients with BUC.

Parameter	Number
Age (y)	
Mean ± SD	57.45 ± 9.08
Range	35–74
Gender	
Male	159 (78.3)
Female	44 (21.7)
Tumor size (cm)	
Mean ± SD	3.31 ± 1.39
Range	0.7–6.1
Multiplicity	
Single	78 (38.4)
Multiple	125 (61.6)
Tumor grade [n (%)]	
Low	66 (32.5)
High	137 (67.5)
cT [n (%)]	
T1-T2	94 (46.3)
T3-T4	109 (53.7)
LNS	
Negative	139 (68.5)
Positive	64 (31.5)

### The expression of PLAGL2 in BUC, positive lymph nodes and normal bladder tissue

**Figure 1:**
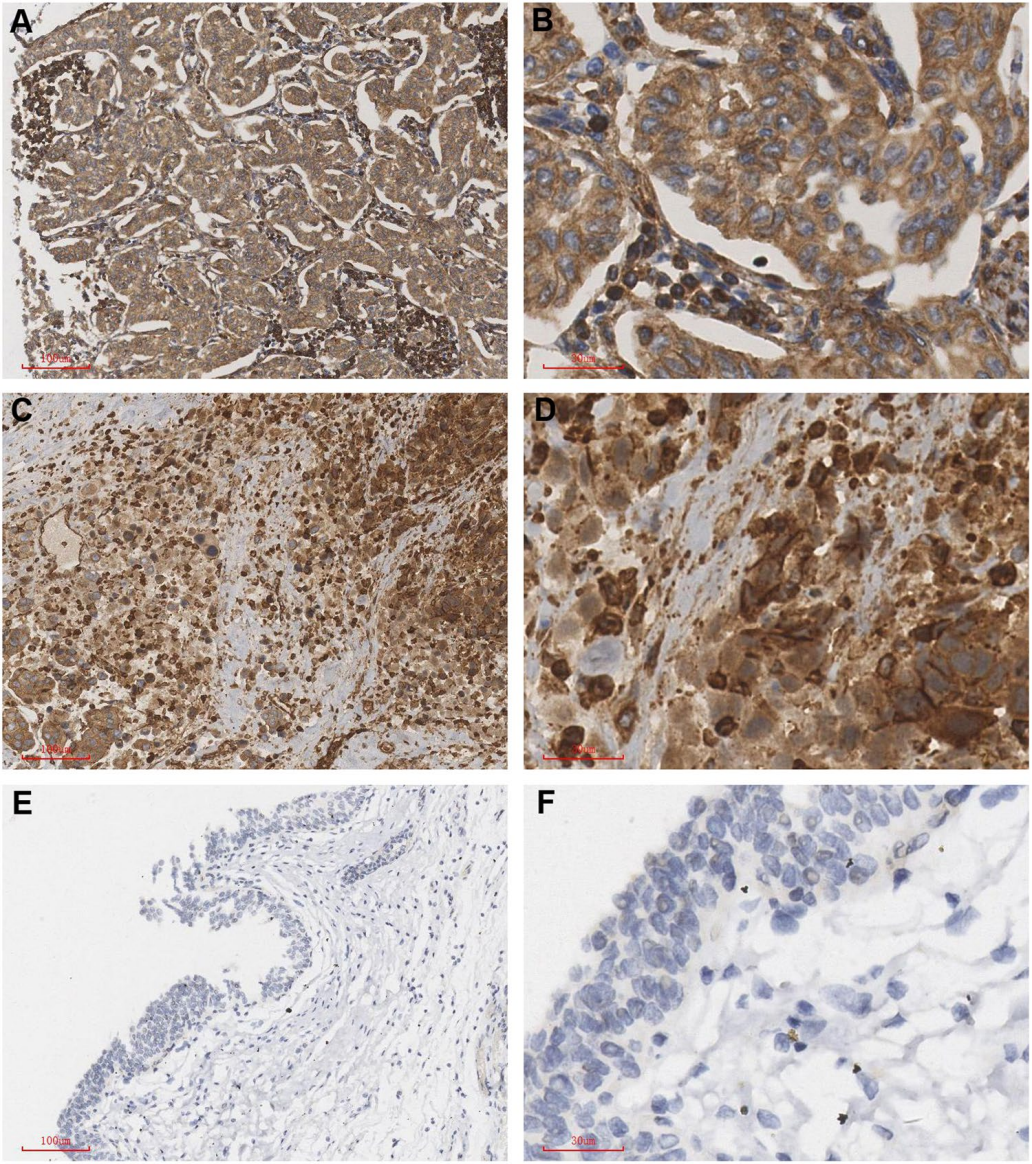
The expression of PLAGL2 in the positive lymph nodes of the bladder cancer was significantly higher than the expression in the primary BUC tissues. The expression of PLAGL2 in BUC tissues was significantly higher than the expression in normal tissue (**A**,**B**) Positive lymph nodes: (**C**,**D**) BUC; (**E**,**F**) Normal bladder tissue; (**A**,**C**,**E**), ×100; (**B**,**D**,**F**), ×400).

PLAGL2 was mainly expressed in the cytoplasm and nucleus of the tumor cells. 64 sections of normal bladder tissues and positive lymph nodes were selected for immunohistochemistry. These sections were also compared to the corresponding primary BUC tissues. The expression rates of PLAGL2 in the positive lymph nodes, BUC, and normal bladder tissues were 89.06%, 76.56% and 21.88%, respectively. The difference between any of the two groups was statistically significant (*P* < 0.05; Table 2, Fig. 1).

**Table 2:** The expression of PLAGL2 in BUC, positive lymph nodes, and normal bladder tissue.

Group	PLAGL2 expression				Positive rate (%)
	−	+	++	+++	
Normal bladder tissue	50	10	4	0	21.88
BUC	15	19	19	11	76.56
Positive lymph nodes	7	6	26	25	89.06

Note: BUC vs. Normal bladder tissue, *P* < 0.05; Positive lymph nodes vs. BUC, *P* < 0.05; *P*ositive lymph nodes vs. Normal bladder tissue, *P* < 0.05.

### Relationship between PLAGL2 expression and pathologic parameters in patients with BUC

**Figure 2:**
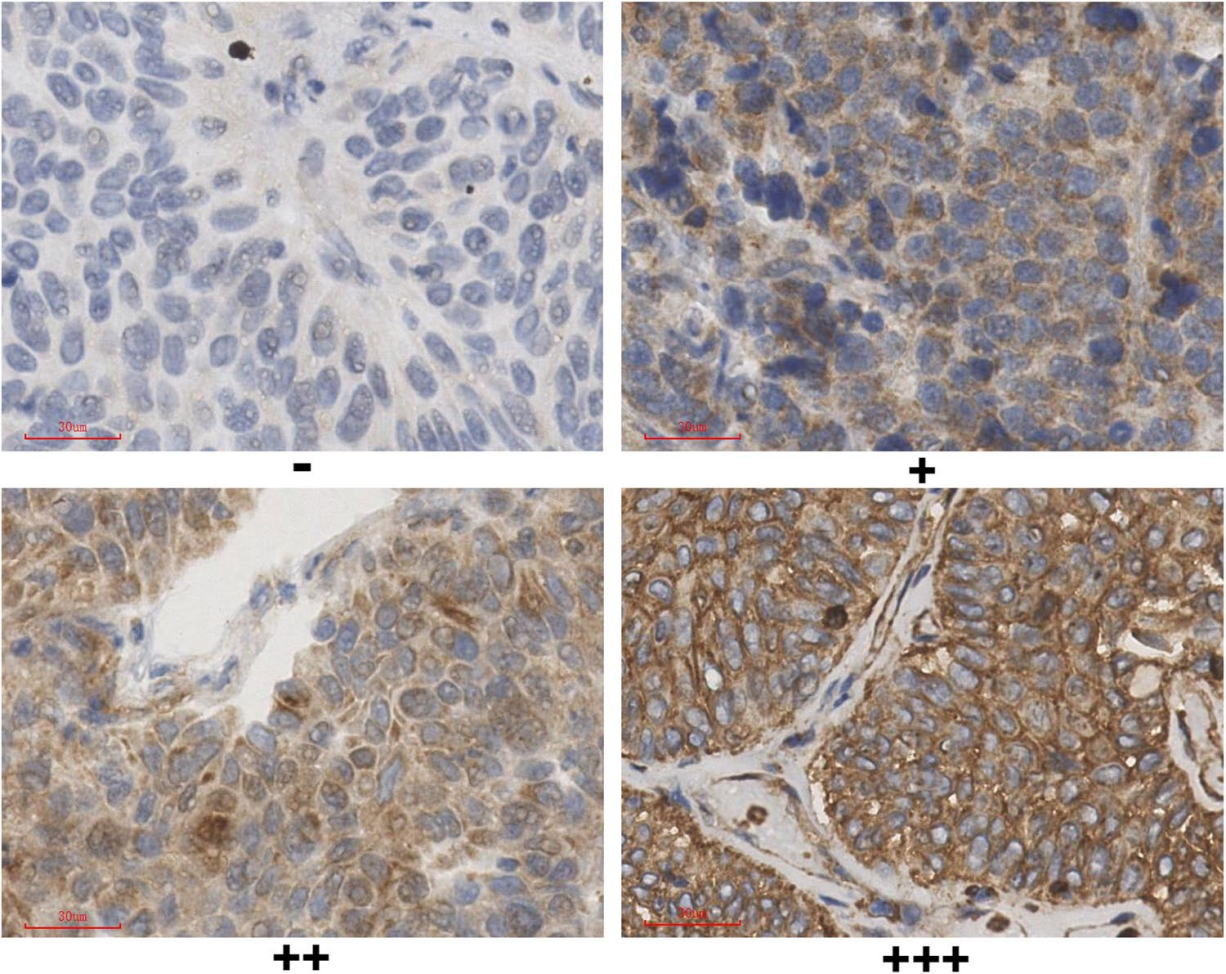
Different expression of PLAGL2 in BUC. Strong staining of PLAGL2 is more common in high-grade, high stage and positive LNS specimens (×400).

The overexpression of PLAGL2 was positively correlated with tumor size, multiplicity of tumors, tumor grade, tumor cT, and LNS, (*P* < 0.05, Table 3). It had no significant relationship to age and sex (*P* > 0.05, Table 3). Strong staining of PLAGL2 was more common in the larger, multiple, high-grade, high stage, and positive lymph node tumor specimens (Fig. 2).

**Table 3:** Relationship between PLAGL2 expression and pathologic parameters in patients with BUC.

Variables	N	PLAGL2 expression				*P* value
		**−**	**+**	**++**	**+++**	
Total, n (%)	203	34 (16.7)	41 (20.2)	73 (36.0)	55 (27.1)	
Age (yrs)						
<60	98 (48.3)	19 (55.9)	18 (43.9)	33 (45.2)	28 (50.9)	0.681
≥60	105 (51.7)	15 (44.1)	23 (56.1)	40 (54.8)	27 (49.1)	
Gender						
Male	159 (78.3)	27 (79.4)	29 (70.7)	59 (80.8)	44 (80.0)	0.622
Female	44 (21.7)	7 (20.6)	12 (29.3)	14 (19.2)	11 (20.0)	
Tumor size (cm)						
<3	90 (44.3)	27 (79.4)	21 (51.2)	26 (35.6)	16 (29.1)	<0.001
≥3	113 (55.7)	7 (20.6)	20 (48.8)	47 (64.4)	39 (70.9)	
Multiplicity						
Single	78 (38.4)	17 (50.0)	9 (22.0)	24 (32.9)	28 (50.9)	0.010
Multiple	125 (61.6)	17 (50.0)	32 (78.0)	49 (67.1)	27 (49.1)	
Tumor grade [n (%)]						
Low	66 (32.5)	16 (47.1)	20 (48.8)	18 (24.7)	12 (21.8)	0.004
High	137 (67.5)	18 (52.9)	21 (51.2)	55 (75.3)	43 (78.2)	
cT [n (%)]						
T1-T2	94 (46.3)	27 (79.4)	24 (58.5)	26 (35.6)	17 (30.9)	<0.001
T3- T4	109 (53.7)	7 (20.6)	17 (41.5)	47 (64.4)	38 (69.1)	
LNS						
Negative	139 (68.5)	28 (82.4)	36 (87.8)	45 (61.6)	30 (54.5)	0.001
Positive	64 (31.5)	6 (17.6)	5 (12.2)	28 (38.4)	25 (45.5)

### The effects of PLAGL2 expression and other clinicopathological parameters on pelvic lymph node metastasis of BUC

Based on subgroup analysis of lymph node metastasis, we found that the pelvic lymph node metastasis of BUC was significantly correlated with tumor size, multiplicity of tumors, tumor grade, tumor cT, and the level of PLAGL2 expression. (*P* < 0.05, Table 4). It was not correlated with the age and gender of the patients (*P* > 0.05, Table 4). Multivariate logistic regression analysis suggested that PLAGL2 overexpression was an independent risk factor for lymph node metastasis of BUC (*P* < 0.05, Table 5).

**Table 4:** Association of clinicopathological features with lymph node metastasis of bladder urothelial carcinoma.

Variables	N	LNS		*P* value
		Negative	Positive	
Total, n (%)	203	139 (68.5)	64 (31.5)	
Age (yrs.)				
<60	98 (48.3)	71 (51.1)	27 (42.2)	0.290
≥60	105 (51.7)	68 (48.9)	37 (57.8)	
Gender				
Male	159 (78.3)	106 (76.3)	53 (82.8)	0.361
Female	44 (21.7)	33 (23.7)	11 (17.2)	
Tumor size (cm)				
<3	90 (44.3)	73 (52.5)	17 (26.6)	0.001
≥3	113 (55.7)	66 (47.5)	47 (73.4)	
Multiplicity				
Single	78 (38.4)	60 (43.2)	18 (28.1)	0.045
Multiple	125 (61.6)	79 (56.8)	46 (71.9)	
Tumor grade [n (%)]				
Low	66 (32.5)	55 (39.6)	11 (17.2)	0.002
High	137 (67.5)	84 (60.4)	53 (82.8)	
cT [n (%)]				
T1-T2	94 (46.3)	77 (55.4)	17 (26.6)	<0.001
T3- T4	109 (53.7)	62 (44.6)	47 (73.4)	
PLAGL2 expression				
−	34 (16.7)	28 (20.1)	6 (9.4)	<0.001
**+**	41 (20.2)	36 (25.9)	5 (7.8)	
**++**	73 (36.0)	45 (32.4)	28 (43.8)	
**+++**	55 (27.1)	30 (21.6)	25 (39.1)

**Table 5:** Multivariable analysis of clinicopathological features for association with lymph node metastasis of BUC.

Variables	OR (95% CI)	*P* value
Tumor size (<3 vs. ≥3)	1.541 (0.717–3.313)	0.268
Multiplicity (Single vs. Multiple)	1.813 (0.884–3.714)	0.104
Tumor grade (Low vs. High)	1.586 (0.684–3.680)	0.283
cT stage (T1-T2 vs. T3- T4)	1.771 (0.831–3.771)	0.138
PLAGL2 (−/+/++/+++)	1.594 (1.100–2.311)	0.014

### Factors influencing recurrence and tumor-free survival rate of BUC patients at 5-year post operation

**Figure 3:**
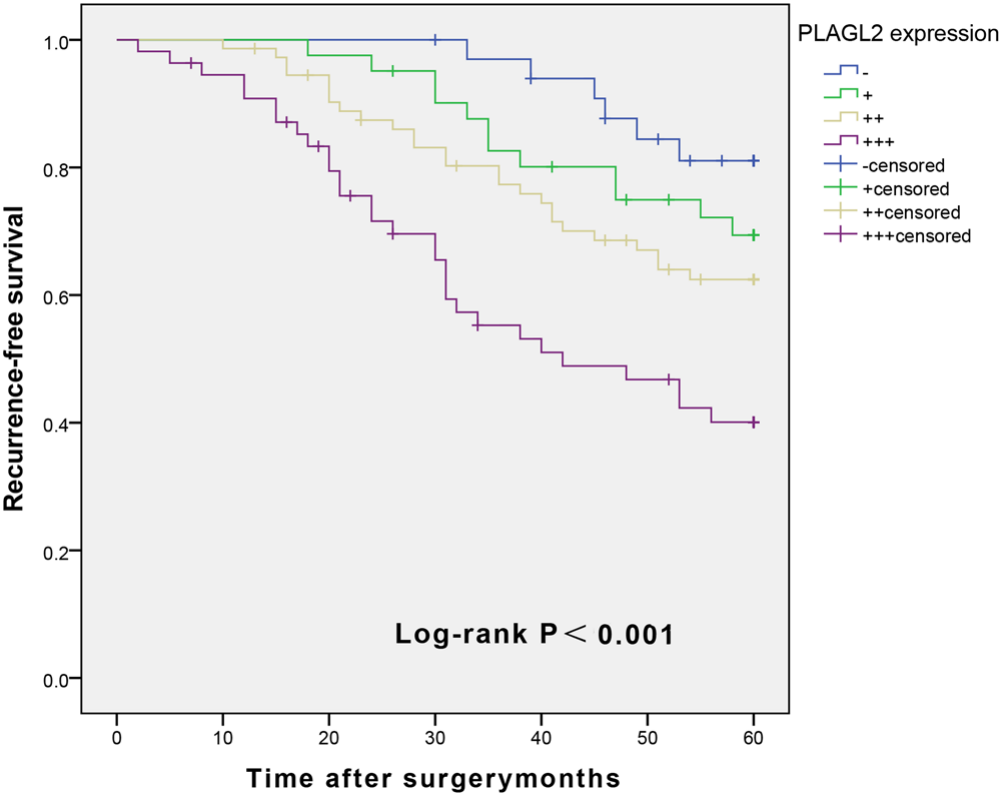
Survival curve of PLAGL2 expression and 5-year RFS rate in patients with BUC after radical cystectomy and pelvic lymphadenectomy (Patients with overexpression of PLAGL2 have a shorter RFS time relative to those with lower expression (log-rank test, *P* < 0.001).

COX singular factor regression analysis showed that tumor size, tumor cT, lymph node metastasis, and the level of PLAGL2 expression were correlated to 5-year RFS of BUC (*P* < 0.05, Table 6). COX multivariate analysis suggested that lymph node metastasis and PLAGL2 overexpression resulted in a shorter RFS for BUC patients (*P* < 0.05, Table 6). The 5-year RFS curve of BUC was tested with Kaplan-Meier survival curve (Fig. 3). It showed that PLAGL2 overexpression had a significantly shorter RFS (*P* < 0.001).

**Table 6:** Univariate and multivariate Cox proportional hazards analyses for the five-year RFS.

Variables	Univariate analysis		Multivariate analysis	
	HR (95% CI)	*P* value	HR (95% CI)	*P* value
Age (yrs.; <60 vs. ≥60)	1.284 (0.812–2.031)	0.285		
Gender (Male vs. Female)	1.379 (0.825–2.304)	0.220		
Tumor size (<3 vs. ≥3)	1.756 (1.089–2.831)	0.021	1.099 (0.655–1.843)	0.722
Multiplicity (Single vs. Multiple)	1.459 (0.901–2.363)	0.125		
Tumor grade (Low vs. High)	1.268 (0.770–2.088)	0.350		
cT stage (T1-T2 vs. T3- T4)	2.193 (1.353–3.553)	0.001	1.447 (0.850–2.464)	0.174
LNS (Negative vs. Positive)	2.438 (1.536–3.869)	<0.001	1.976 (1.228–3.178)	0.005
PLAGL2 (−/+/++/+++)	1.703 (1.325–2.190)	<0.001	1.578 (1.205–2.066)	0.001

### Factors influencing the disease-free OS rate of BUC patients at 5-year post operation

**Figure 4:**
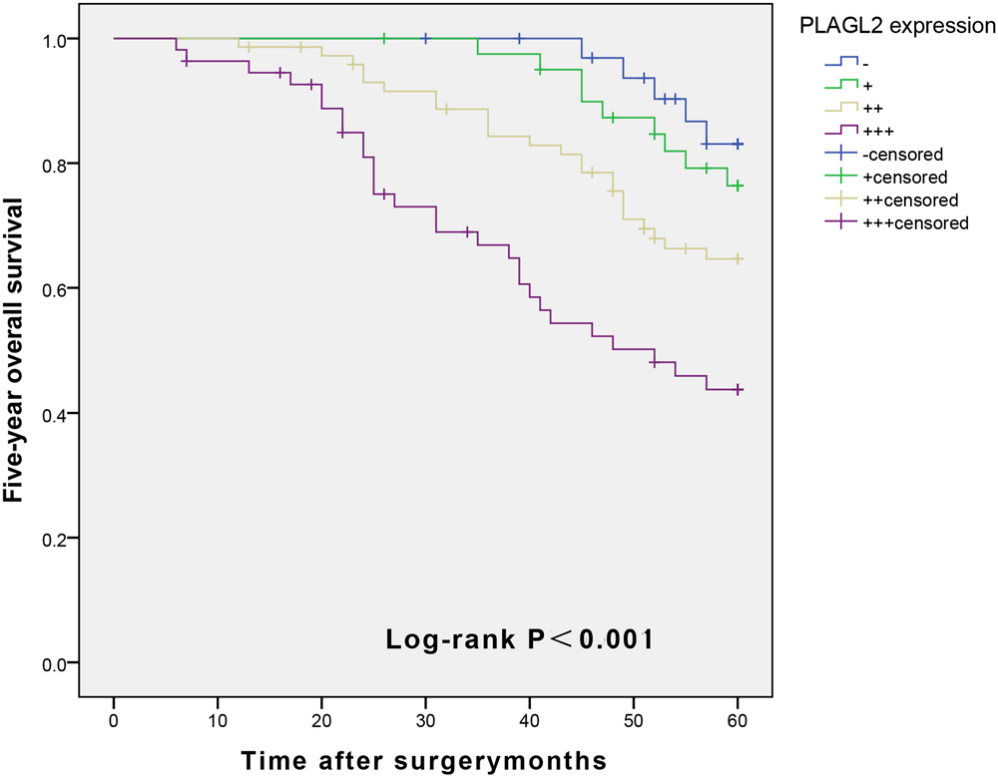
Survival curve of PLAGL2 expression and 5-year OS in patients with BUC after radical cystectomy and pelvic lymphadenectomy (Patients with overexpression of PLAGL2 have a shorter five-year OS relative to those with lower expression (log-rank test, *P* < 0.001).

COX single factor regression analysis showed that tumor size, cT, lymph node metastasis, and overexpression of PLAGL2 were correlated with disease-free OS of BUC (*P* < 0.05, Table 7). COX multivariate analysis showed that a high cT stage, lymph node metastasis, and overexpression of PLAGL2 were predictors of a shorter 5-year survival time (*P* < 0.05, Table 7). The 5-year OS curve of BUC was tested with the Kaplan-Meier curve (Fig. 4). It showed that PLAGL2 overexpression was associated with a 5-year survival time, which is significantly shorter than the survival time of patients with lower expression *(P* < 0.001).

**Table 7:** Univariate and multivariate Cox proportional hazards analyses for the five-year OS.

Variables	Univariate analysis		Multivariate analysis	
	HR (95% CI)	*P* value	HR (95% CI)	*P* value
Age (yrs.; <60 vs. ≥60)	1.510 (0.924–2.466)	0.100		
Gender (Male vs. >25)	1.448 (0.850–2.468)	0.173		
Tumor diameter (<3 vs. ≥3)	1.889 (1.132–3.151)	0.015	1.050 (0.607–1.816)	0.861
Multiplicity (Single vs. Multiple)	1.550 (0.923–2.602)	0.098		
Tumor grade (Low vs. High)	1.440 (0.838–2.476)	0.187		
cT stage (T1-T2 vs. T3- T4)	2.777 (1.629–4.735)	<0.001	1.801 (1.012–3.204)	0.045
LNS (Negative vs. Positive)	3.042 (1.873–4.940)	<0.001	2.438 (1.483–4.008)	<0.001
PLAGL2 (−/+/++/+++)	1.852 (1.408–2.436)	<0.001	1.717 (1.276–2.309)	<0.001

## Discussion

BUC is the most common malignant tumors of the urinary tract and about 30% of BUC are invasive tumors^10^. Lymphatics is the most important pathway for metastasis. The presence of lymph node metastasis is a predictor for a poor prognosis^11^. The polymorphic adenoma gene-like protein (PLAGL) of the zinc finger protein gene is extensively found in the mammalian genome. PLAGL, PLAGLl and PLAGL2 constitute the complete family. The zinc finger protein family is commonly involved in a variety of physiological and pathological processes in the form of nucleoprotein transcription factors. Many studies have shown overexpression of PLAGL2 in various tumor tissues such as lung cancer, gastric cancer, colorectal adenocarcinoma, and prostate cancer^6,8,9^. In our study, the positive rate of PLAGL2 expression in the bladder cancer tissue was 83.3%; of which weak positive rate (+) was 20.2%, intermediate positive rate (++) was 36.0%, and strong positive rate (+++) was 27.1%. On the other hand, the positive rate of PLAGL2 expression was 21.88% in the 64 normal bladder tissues; all of which was either weak positive or intermediate positive. This difference was statistically significant. We have thus confirmed for the first time the overexpression of PLAGL2 in the BUC.

We also showed that the overexpression of PLAGL2 is correlated with tumor size, multiplicity of tumors, pathological grade, tumor cT, and lymph node metastasis. COX multivariate analysis suggested that lymph node metastasis and overexpression of PLAGL2 are important factors for diminishing the 5-years postoperative RFS and OS of the BUC patients. Braud *et al*.^12^ found that the survival time of patients with regional metastasis after radical cystectomy and pelvic lymphadenectomy was 20% lower than those without. Karakiewicz *et al*.^13^ reported that tumor grade, tumor cT, and the presence of carcinoma *in situ* are independent predicators of lymph node metastasis and ultimately the survival of the patients.

The cT, pathological grade, and lymph node metastasis of bladder cancer are important predictors for prognoses^14,15^. Hensen *et al*.^7^ have shown that the overexpression of PLAGL2 could cause tumor formation. PLAGL2 overexpression was correlated with the typical manifestations of malignant transformation. Abdollahi^16^ found that the overexpression of PLAGL2 not only caused acute granulocytic leukemia in mice, but was also found in 20% of acute myeloid leukemia patients^16^. Other studies have shown that PLAGL2 was highly expressed in a variety of tumors and promoted tumor progression and metastasis^6,17^. Bladder urothelial carcinoma (BUC) with lymph node metastasis often denotes a poor prognosis. In clinical practice, the existence of lymph node metastasis prior to surgical resection relies on MRI and/or CT studies. However, MRI or CT studies are often equivocal in identifying small lymph node metastases or lymphadenopathy caused by inflammatory conditions. Our study found that the risk of lymph node metastasis in PLAGL2-positive bladder cancer patients was 1.661 times higher than that of PLAGL2-negative bladder cancer patients. Thus PLAGL2 overexpression can be an independent predictor for lymph node metastasis.

The relationships of PLAGL2 expression with lymph node metastasis and patient survival have great clinical significance. Currently pelvic lymphadenectomy is routinely performed with radical cystectomy for high-risk bladder tumors. Some experts advocated extended pelvic lymph node dissection to further improve prognoses^18^. However, extending lymphadenectomy may lead to longer operations and increased morbidities. If there is a reliable preoperative predictor for lymph node metastasis, this predictor could provide guidance for the pelvic lymph node dissection. The results of our study showed that PLAGL2 could be used as an independent predictor for pelvic lymph node metastasis. Furthermore, PLAGL2 could be used as a predictor for recurrence-free and overall 5-year survival of bladder cancer patients.

Cancer biomarkers are indicators that can objectively evaluate and indicate biological processes, pathological processes, and pharmacological responses to interventions, however, clinically useful cancer biomarkers remain rare, because cancer is a complex disease^19^. Thus far, no effective biomarkers have yet been found in bladder cancer. Our results show that PLAGL2 can be used as a cancer hallmarks for bladder cancer. And cancer hallmarks can enable tumor growth and metastasis dissemination, the collection of these hallmarks could help in understanding of the mechanisms by which invading cells give rise to recurrent tumors and the effect of adjuvant therapeutics have on their evolution which will facilitate the development of new strategies to delay or prevent recurrence and malignant tumor progression^20^. Through the detection of cancer hallmarks, it is possible to judge the course of the disease, predict the survival of the patient, as well as the efficacy and prognosis. And the continuous exploration of cancer hallmarks and the gradual realization of individualization of treatment have become the current research hotspots^21^.

The weaknesses of this study include the following. First, it was a retrospective study, so it had inherent bias. Second, the comparison of the tissues were all from patients with BUC rather than normal patients. Comparison with normal bladder tissue from patients without BUC would be more precise. However, this approach would not be practical. The specific mechanism of PLAGL2 is still unknown. Further work is needed to elucidate the role and function of PLAGL2 in tumor genesis.

## Conclusion

PLAGL2 expression can be used as an independent predictor for pelvic lymph node metastasis in BUC. Overexpression of PLAGL2 was found to correlate with tumor size, number, grade, and stage. PLAGL2 expression can serve as predictor for patients’ 5-year RFS and OS after radical cystectomy with pelvic lymphadenectomy.

## Materials and Methods

### Tissue specimen and data collection

203 consecutive surgical specimens from bladder cancer patients after radical cystectomy with standard lymph node dissections were collected from the Zhuzhou Central Hospital from January 2007 to January 2011, (The extent of standard lymph node dissection is templated as following: lateral to femerogenital nerve, medial to obturator nerve, superior extent at iliac vessel bifurcation, inferior extent at the distal iliac vein.) and all patients were informed consent for study participation. Specimens were fixed with 4% formaldehyde and embedded in paraffin. Pathological examination confirmed that tumors were BUC. Patients who received radiotherapy and chemotherapy before surgery were excluded. In addition, 64 normal bladder sections and 64 positive lymph nodes were selected as comparison groups. Clinical data collected included the age, gender, tumor size, multiplicity of tumors, tumor grade, tumor clinical stage (cT), and lymph node status (LNS) (Table 1).

### Construction of tissue microarray

The paraffin-embedded specimens of BUC, positive lymph nodes, and normal bladder tissues were sectioned for hematoxylin and eosin (H&E) staining. Diagnosis was confirmed and representative areas of the tissues were identified for microarray processing. Two 2 × 2 mm tissue cores from each specimen were taken from the corresponding paraffin blocks. Total 5 × 8 tissue cores were placed into each of the receptacle blocks. The microarray blocks were then sliced to a thickness of 4 μm.

### Immunohistochemistry

These sections were conventionally de-waxed and rehydrated. Antigen retrieval was performed using a high-pressure cooker at 95 °C in 0.1 M citric acid buffer (pH 5.0 Fuzhou Maixin Biotech.Co., Ltd., Fuzhou, China). The tissue microarrays were then incubated with 3% H_2_O_2_ for 10 minutes to block endogenous peroxidase activity, followed by normal goat serum at room temperature. Next, the tissue microarrays were incubated with a rabbit monoclonal anti-PLAGL2 antibody (primary antibody, Abcam Company, UK) at a dilution of 1:800 at 4 °C overnight followed by incubation with DAKO ChemMate EnVision (secondary antibody, DAKO Company, USA) at 37 °C for 30 minutes. The sections were subjected to staining with 3,30-diaminobenzidine (DAB) solution and briefly counterstained with hematoxylin. Last, the sections were dehydrated with ethanol, mounted with a cover slip, and examined with a microscope.

### Judgment of the results

The immunologically stained microarray sections were blindly scored by two senior pathologists. Positive expression of PLAGL2 was identified by the appearance of brown granules in cytoplasm and nucleus. Five representative high-power fields from each slide were examined. 100 cells in each field were evaluated. The proportion of positive stained cells and staining intensity were scored. The following criteria were used: ① Staining intensity (I) was recorded as: no staining, 0; weak staining intensity (light yellow or only individual cells were yellow to brown stained), 1; medium staining intensity (between the first two), 2; and strong staining intensity (yellow to brown stained), 3. ② The proportion (P) of positive cells was recorded as 0, <5%; 1, 5% to 25%; 2, 26% to 50%; 3, 51% to 75%; and 4, >75%. For total points, ① + ②, 0 is negative (−), 1–2 is weak positive (+), 3–5 is positive (++), 6–7 is strong positive (+++)^22^. Any discrepancy in the scoring was resolved by discussion between the pathologists. And all methods were performed in accordance with the relevant guidelines and regulations.

### Statistical methods

Statistical analysis was performed using the SPSS 22.0 statistical software package. Qualitative data were analyzed by independent sample chi-square test or by the Fisher exact test. The predictive value of lymph node metastasis was investigated using Logistic regression analysis. The relationships of PLAGL2 expression with the 5-year recurrence-free survival (RFS) and the overall survival (OS) of BUC were estimated by Kaplan-Meier survival curve and Log-rank statistical method. The singular and multivariate analyses of the 5-year RFS and the OS of BUC were evaluated using the COX proportional hazards regression model. *P* < 0.05 was considered to be statistically significant.

## References

[1] Ferlay J (2015). Cancer incidence and mortality worldwide: sources, methods and major patterns in GLOBOCAN 2012. International journal of cancer.

[2] Pang C, Guan Y, Li H, Chen W, Zhu G (2016). Urologic cancer in China. Japanese journal of clinical oncology.

[3] Simms MS, Mann G, Kockelbergh RC, Mellon JK (2005). The management of lymph node metastasis from bladder cancer. European journal of surgical oncology: the journal of the European Society of Surgical Oncology and the British Association of Surgical Oncology.

[4] Kas K, Voz ML, Hensen K, Meyen E, Van de Ven WJ (1998). Transcriptional activation capacity of the novel PLAG family of zinc finger proteins. The Journal of biological chemistry.

[5] Zheng H (2010). PLAGL2 regulates Wnt signaling to impede differentiation in neural stem cells and gliomas. Cancer cell.

[6] Yang YS, Yang MC, Weissler JC (2011). Pleiomorphic adenoma gene-like 2 expression is associated with the development of lung adenocarcinoma and emphysema. Lung cancer (Amsterdam, Netherlands).

[7] Hensen K, Van Valckenborgh IC, Kas K, Van de Ven WJ, Voz ML (2002). The tumorigenic diversity of the three PLAG family members is associated with different DNA binding capacities. Cancer research.

[8] Liu B (2014). The role of pleomorphic adenoma gene-like 2 in gastrointestinal cancer development, progression, and prognosis. International journal of clinical and experimental pathology.

[9] Guo J, Wang M, Wang Z, Liu X (2016). Overexpression of Pleomorphic Adenoma Gene-Like 2 Is a Novel Poor Prognostic Marker of Prostate Cancer. PloS one.

[10] Karl A (2009). The impact of lymphadenectomy and lymph node metastasis on the outcomes of radical cystectomy for bladder cancer. European urology.

[11] Ahn TS (2015). Extracapsular Extension of Pelvic Lymph Node Metastasis is an Independent Prognostic Factor in Bladder Cancer: A Systematic Review and Meta-analysis. Ann Surg Oncol.

[12] Braud G, Battisti S, Karam G, Bouchot O, Rigaud J (2008). Prognostic value of lymph node dissections in bladder cancer treated with radical cystectomy. Progres en urologie: journal de l’Association francaise d’urologie et de la Societe francaise d’urologie.

[13] Karakiewicz, P. I. *et al*. Precystectomy nomogram for prediction of advanced bladder cancer stage. *European urology* **50**, 1254–1260; discussion 1261–1252 10.1016/j.eururo.2006.06.010 (2006). 10.1016/j.eururo.2006.06.010 16831511

[14] Lughezzani G (2010). Adenocarcinoma versus urothelial carcinoma of the urinary bladder: comparison between pathologic stage at radical cystectomy and cancer-specific mortality. Urology.

[15] Schultz L (2010). Expression status and prognostic significance of mammalian target of rapamycin pathway members in urothelial carcinoma of urinary bladder after cystectomy. Cancer.

[16] Abdollahi A (2007). LOT1 (ZAC1/PLAGL1) and its family members: mechanisms and functions. Journal of cellular physiology.

[17] Yang MC, Weissler JC, Terada LS, Deng F, Yang YS (2005). Pleiomorphic adenoma gene-like-2, a zinc finger protein, transactivates the surfactant protein-C promoter. American journal of respiratory cell and molecular biology.

[18] Hurle R, Naspro R (2010). Pelvic lymphadenectomy during radical cystectomy: a review of the literature. Surg Oncol.

[19] Gao S (2016). Identification and Construction of Combinatory Cancer Hallmark-Based Gene Signature Sets to Predict Recurrence and Chemotherapy Benefit in Stage II Colorectal Cancer. JAMA oncology.

[20] Li J (2010). Identification of high-quality cancer prognostic markers and metastasis network modules. Nature communications.

[21] Wang E (2015). Predictive genomics: a cancer hallmark network framework for predicting tumor clinical phenotypes using genome sequencing data. Seminars in cancer biology.

[22] Ma HQ (2009). Decreased expression of Neurensin-2 correlates with poor prognosis in hepatocellular carcinoma. World journal of gastroenterology.

